# Neuroscience-based psychotherapy: A position paper

**DOI:** 10.3389/fpsyg.2023.1101044

**Published:** 2023-02-13

**Authors:** Davide Maria Cammisuli, Gianluca Castelnuovo

**Affiliations:** ^1^Department of Psychology, Catholic University, Milan, Italy; ^2^Psychology Research Laboratory, Istituto Auxologico Italiano IRCCS, Milan, Italy

**Keywords:** traumatic memories, attachment theory, cognitive psychopathology, empathy, neuroimaging, somatoform disorders, neuroscience, psychotherapy

## Abstract

In the recent years, discoveries in neuroscience have greatly impacted upon the need to modify therapeutic practice starting from the evidence showing some cerebral mechanisms capable of coping with mental health crisis and traumatic events of the individual's life history by redesigning the narrative plot and the person's sense of the Self. The emerging dialogue between neuroscience and psychotherapy is increasingly intense and modern psychotherapy cannot ignore the heritage deriving from studies about neuropsychological modification of memory traces, neurobiology of attachment theory, cognitive mechanisms involved in psychopathology, neurophysiology of human empathy, neuroimaging evidence about psychotherapeutic treatment, and somatoform disorders connecting the brain and the body. In the present article, we critically examined sectorial literature and claimed that psychotherapy has to referred to a neuroscience-based approach in order to adopt the most tailored interventions for specific groups of patients or therapy settings. We also provided recommendations for care implementation in clinical practice and illustrated challenges of future research.

## 1. Introduction

In the second half of the 19th century, schools in psychotherapy were born according to different epistemological constructs and methods, with the aim of removing trauma, modifying symptoms of affective nature, and promoting the development of a healthy personality (Janiri et al., 2009). Psychotherapy can be defined in relation to three main elements, that is a helping relationship established after the parent–child one, the creation of a secure base from which the therapist influences the patient through psychological techniques, and a patient who has the capacity to benefit from such an experience (Strupp, 1978). However, Freud himself first recognized the limit of the only adoption of a pure introspective approach, stating that *the deficiency in [our] description would probably vanish if we were already in a position to replace the psychological terms by physiological or chemical ones* (Freud, 1920, p. 60). He first endeavored to establish the restraint of the “*talking cure*” and to clarify that the progress of what we currently name as “neuroscience” would have achieved the result to bridge this gap (Solms and Turnbull, 2018).

With the advent of neuroscience, a highly integrated discipline, as well as astrophysics, knowledge in the fields of molecular and cellular biology, biochemistry, neurophysiology, neuroradiology, and general and experimental psychology, has harmoniously merged, creating a body of evidence of which psychotherapy has to benefit. One of the most fruitful experiences that determined a new approach to scientific research in brain science was that of Francis O. Schmitt, who set up the Neuroscience Research Program (NRP) at the Massachusetts Institute of Technology (MIT) in 1962. He intended the NRP as a research field connecting physical, biological, and neural science for a better understanding of the liaison between mind, brain, and behavior (Adelman, 2010). Neuroscience grew rapidly from this time point on as scientists from all areas of the life sciences rapidly moved into the field. This process culminated with the foundation of the Society for Neuroscience (SfN) in 1969 (Adelman, 2010).

An important turnover in the process of the emerging dialogue between psychotherapy and neuroscience was undoubtedly the scientific contribution of Kandel (1998, 2012), who went on to the Nobel Prize for medicine/physiology in 2000 by providing a breakthrough perspective on how biology has influenced modern psychiatry, especially on how memory and learning processes can explain behavior and its disorder and implications of the neurobiological research in psychotherapy. Kandel's description of the influence of culture on genetics and of the mechanism of “reconsolidation” of memory emphasizes the importance of psychotherapy for mental disorders. Particularly, practicing psychotherapy as a strategic and significant influence on the patient's living environment can be particularly effective in modeling gene expression and promoting behavioral modifications (Etkin et al., 2005).

Beyond different treatment approaches (i.e., psychoanalysis and psychodynamic therapies, cognitive–behavioral therapy, humanistic therapy, family and system psychotherapy, interpersonal psychotherapy, and integrative or holistic therapies) (Roth and Fonagy, 2005), psychotherapy involves common elements, such as verbal and non-verbal exchanges as interactions between therapist and patient, therapeutic alliance, empathy, resilience to trauma, cognitive restructuring, and new learning (Gabbard, 2009) that we currently believe psychotherapists have to read under the magnifying glass of neuroscience. Currently, on at least six major study areas, some of which already pointed out in Janiri et al. (2009), neurosciences have provided valuable contributions to the understanding of the neurobiological substrate of brain changes useful for psychotherapy practice: memory of trauma, neurobiological correlates of human attachment, mirror neurons system and theory of mind (ToM), brain modifications after psychotherapeutic treatment, and somatic symptoms and disorders. To let the scientific audience better focus on these areas, we provided a summary table for each section of the manuscript reporting major recommendations for mental health practitioners. We firmly affirm that psychotherapists in the modern era can no longer ignore discoveries in the field of neuroscience and have to learn from them in order to implement their clinical practice.

## 2. Psychotherapy of traumatic memories

There are organic molecular mechanisms that are fundamental for the establishment of long-term memories. For instance, the corticosterone hormone, which corresponds to the cortisol in humans, released after a stressor input in animals, rapidly interacts with growth factors produced in the brain, in particular with the brain-derived neurotrophic factor (BDNF), a neurotrophin essential for long-term synaptic plasticity (Alberini, 2009). These mechanisms occur in the medial temporal lobe, specifically in the hippocampus, which plays a central part in long-term memory formation (Johnston and Amaral, 2004). Peripheral BDFN levels are lower in psychiatric disorders (Boulle et al., 2012), and a recent systematic review has shown that BDNF seems to present variations after psychotherapy, especially in patients with bulimia, post-traumatic stress disorder (PTSD), insomnia, and borderline personality disorder, with a reduction in symptomatology (Claudino et al., 2020).

Memory represents a system of brain networks that presides over different functionally coordinated and anatomically independent cognitive mechanisms sharing the ability to store information (Squire, 2004). Particularly, explicit (or declarative) memory is involved in the psychotherapeutic process. As reported in Freud-Fliess Letters (1887–1904), *Our psychic mechanism is formed, is created through a process of stratifications. The material that is present as memory or trace of memory undergoing continuous reconstruction, a renewal with respect to the present, to new circumstances. Memory is not present once, it does not remain as a single trace but continues to renew itself* (Albertini, 2014). Research in neuroscience has documented that the storage of memories is allowed in relation to a certain level of stress (Schwabe et al., 2012). When the stress level is relatively moderate, memories are formed and retained, and when the stress becomes too prolonged or intense, it generates a negative effect on memories that can even be lost, like in the case of trauma (Alberini, 2011). Moreover, regardless of its valence (i.e., positive *vs*. negative), the intensity of an emotion experienced during an original event increases the likelihood that the memory will be recalled later. Scientific evidence has shown that an increased arousal results in a more augmented physiological interaction between the amygdala and the hippocampus, leading to an enhanced encoding and long-term consolidation of information (Lane et al., 2015).

Starting from these findings, it is possible to advance hypotheses on the mechanisms that take place in the room of the psychotherapist when memories are remembered by the patient, precisely traumatic ones. Reconsolidation mechanisms would offer the opportunity to restore memories with a different emotional level, that is, lower stress intensity and greater control. At a molecular level, memory consolidation of an experienced event (i.e., long-term memory) is formed as synaptic connections within a set of neurons (Gallistel and Matzel, 2013). To this end, an experienced event induces a neuronal depolarization and an influx of intracellular Ca^2+^, which initiates a downstream molecular cascade that results in the transcription and translation of plasticity-related proteins (PRPs) inducing changes in neuronal networks as remodeled synaptic connections (Sekeres et al., 2017). The activity among synaptic connections leads to the development of “memory engram” requiring a period of quiescence to be stabilized that can be potentially interrupted by protein-synthesis inhibition or interference from new learning (Sekeres et al., 2017). The multiple trace theory (MTT) (Nadel and Moscovitch, 1997; Moscovitch and Nadel, 1999) offers insight into how the repeated recollection of prior events can transform memory representations. Accordingly, the establishment of long-term memory involves a long interaction between hippocampal regions of the medial temporal lobes and medial prefrontal cortex. MTT proposes that each time an episodic memory is elicited by active retrieval or recollection, an update trace is created, incorporating information from the old trace and new recall. According to Albertini (2014), *remembering with the psychotherapist* creates the possibility of associating old reactivated memories with new ones moving from painful experiences faced during treatment. In the presence of new elements and of the support given by the therapist, restored memory has different qualities (i.e., less traumatic and emotionally intense). MTT supports that memories are not the same record of the original event but undergo updating and reshaping as they age and when they are altered by recollection during psychotherapy (Lane et al., 2015). The psychotherapeutic process promotes the possibility of associating old memories reactivated with new experiences of the present, thus soliciting a real behavioral change (Lane, 2020).

A number of psychotherapeutic techniques are considered in the emerging field of “memory therapeutics” including accelerated resolution therapy, rewind techniques, cognitive restructuring, and imagery modification. They aim to help the patient to feel less distressing sensations during memory reactivation, achieve a novel perspective about past negative events, rescript a different ending, and transform trauma using metaphors (Waits and Hoge, 2018). Since memory traces of traumatic events can be weakened, it is helpful to underline the influence of experiences made during psychotherapeutic treatment. Specifically, a distinct type of successful traumatic memory processing in PTSD psychotherapy includes threat processing by intensive imaginal memory retrieval through the support of the therapist for the reappraisal of the event, self-referential reflection on associated beliefs and emotions, and memories retelling (Ford, 2018).

### Key points

- The BDNF—which is related to synaptic plasticity—seems to be low in psychiatric disorders;
- A moderate level of stress is necessary for memory storage;
- Memory reconsolidation supports the psychotherapeutic process for the creation of alternative ways of behaving; and
- Psychotherapy of traumatic memories consists of specific techniques in the case of PTSD.

## 3. Neurobiology of attachment therapy

According to Holmes (1993), attachment theory provides a psychological grounding that is applicable to all forms of psychotherapy in terms of a secure base (i.e., consistency, regularity, and reliability, a combination of warmth and firm boundaries), emersion of autobiographical competence (i.e., a secure attachment in therapy enables the patient to tell a different story about himself/herself), ability in processing affect (i.e., primitive emotions aroused in therapy are modulated by the attunement of the therapist and become manageable for the patient), and coping with loss (i.e., the expression of repressed pain or the modulation of unprocessed angry is an important part of each psychotherapeutic treatment). Attitudes and behavioral modifications of parents during the maternal gestational period, childbirth, breastfeeding, and in the first 3 years of the child's life imply a synergy between hormonal and neurochemical systems and lead to changes in brain structure and functionality, stimulating or inhibiting certain brain areas (Kim, 2016; Grumi et al., 2021). Early life experiences with the own caretakers have a huge impact on the child's brain development because repeated relationship patterns shape undifferentiated neurons into coherent firing networks supporting specific cognitive-affective brain structures (Schore, 2003; Kandel et al., 2013; Grawe, 2017; Siegel, 2020). The attachment relationship between the baby and the primary caretakers represents the link between the neurobiological programming of brain development and early care experiences. The child's bond to caregivers is implicated in the development of the right hemisphere and of specific brain areas (i.e., prefrontal cortex, orbitofrontal cortex, and limbic system) conditioning interpersonal relationships. In fact, such brain areas are involved in the processing and expression of emotional information and influence affective states' modulation and decoding of facial expressions, gestures, and prosody (Etkin et al., 2015). Remarkably, starting from a neuropsychoanalysis perspective, Schore (2022) contended that the right brain represents the psychobiological substrate of the unconscious human mind, as first described by Sigmund Freud, able to process emotional stimuli with implicit attention outside the role of awareness. Early relational experiences with caretakers are transferred into the psychotherapeutic process. Maternal and paternal mental illnesses in the perinatal period negatively impacting the interaction with the baby represent risk factors for the development of the parenting style because they may inhibit the abovementioned child's brain areas (Stein et al., 2014; Lautarescu et al., 2020). Conversely, when parental care contemplates the growth needs of the child, and neuronal networks conform to ensure positive responses to the environment, adults presenting with secure attachment show higher levels of reflective functioning and mature defenses that serve in better regulating affective states than those with insecure attachment (Tanzilli et al., 2021).

### 3.1. Perinatal psychotherapy

With the acronym Perinatal Mood and Anxiety Disorder (PMAD), a consensus in the scientific community has been obtained about the need to go beyond a focus on postpartum depression in the perinatal period toward a more comprehensive mother' symptomatology, including the spectrum of depressive and anxiety disorders, the obsessive–compulsive disorder, the PTSD and the puerperal psychosis (Byrnes, 2018). Furthermore, fathers may display anxiety disorders, somatic symptoms, hypochondria, substance abuse, and behavioral reactivity as maladaptive responses to paternity (Baldoni and Giannotti, 2020). To address these issues, perinatal psychotherapy assumes a fundamental part in caretakers' health regarding manifestations of mental suffering, starting from the multifactorial etiopathogenetic origin of maternal and parental mental illnesses, including neurobiological and hormonal modifications due to childbirth. The activation of the superior temporal sulcus, the amygdala, the right inferior frontal gyrus, and the insula is particularly sensitive to changes in hormonal levels involved in parental care, including augmentation in oxytocin and vasopressin levels in both parents and decrement in testosterone and estradiol for men (Abraham et al., 2014; Witteman et al., 2019). Interesting advances in psychotherapy research have recently highlighted the role of some hormones, such as cortisol and oxytocin, in mediating the relationship between the therapist and the patient, too. The cortisol, or the stress hormone representing the final product of the hypothalamic–pituitary–adrenal axis, is secreted in psychosocial stress contexts (Dickerson and Kemeny, 2004) and is capable of modulating the emotional experience influencing the affect. For instance, higher cortisol levels are associated with significant increases in negative affect in patients with major depressive disorder (MDD) (Booij et al., 2016). Levi et al. (2021) have studied the extent to which therapist's cortisol modulates patient's affect during psychodynamic psychotherapy of MDD by collecting salivary samples before and after specified sessions and data from patient's retrospective reports of in-session affect. The study provided initial evidence that the positive or negative affect of the patient is mediated by the therapist's changes in cortisol levels, supporting the importance of a special social support context built by the interpersonal relationship as an empathic response to the stress manifested by depressed patients. When in a safe situation, a person profoundly perceives his/her engagement, the oxytocin may also be released, supporting reciprocity, empathy, compassion, and synchronized behavior and characterizing parent–infant relationship (Schneiderman et al., 2012; Feldman, 2017). Oxytocin, as the neurohormone associated with care behavior, is implicated in the pathophysiology of MDD in humans (Engel et al., 2019), too, and it is a potential candidate for explaining treatment outcomes in relation to patient–therapist synchrony. Zilcha-Mano et al. (2020) have demonstrated that psychotherapeutic treatment is effective when patients suffering from MDD and therapists are biologically synchronized in oxytocin levels change, giving cutting-edge directions for future research. Starting from these preliminary findings, we think that psychotherapists working on perinatal mental health should reflect on how they build a supportive context and place themselves in sync with the patient since this can actually affect his/her affective state with significant implications for the child's care.

### 3.2. Couple psychotherapy

The integration of the attachment theory with neuroscience also poses a relevant application in the psychotherapy of couples about the dyadic affect regulation during treatment. The goal of neuroscience-based psychotherapy in this specific setting is to help each partner understand his/her part in altering the relationship, starting from personal attachment needs and psychophysiological reactions elicited in the communication with the significant other. By understanding how each partner's nervous reaction is affected by emotional reverberation triggered in the couple's interaction, partners can be treated in psychotherapy to recreate greater emotional control and a secure relationship base (Goldstein and Thau, 2004). When a couple's attachment schema is breached, partners may seek treatment, and psychotherapy plays a pivotal role in restoring a balance between them. Particularly, they should be encouraged to become progressively aware of their unconscious, implicit memories driving personal communication patterns (Schore, 2003). In this way, when partners get into a crisis and start psychotherapy, they are stimulated to progressively recognize the subcortical emotional system they have constructed during the love affair in order to rebuild it in a creative and positive manner. The therapist has to be able to normalize conflictual states by soliciting the couple to pay attention to autonomic responses to dangerous, frightening, and disruptive stimuli, which may be conveyed in the communication between partners, in order to control them and promote assertiveness.

### Key points

- An attachment-based approach in psychotherapy looks at the connection between the infant's primary experiences and caregivers' responses;
- The development of the right hemisphere and of the prefrontal cortex, orbitofrontal cortex, and limbic system is influenced by such a relationship in childhood;
- Perinatal psychotherapists should pay attention to neurochemical and hormonal modifications in new parents;
- The awareness of the subcortical emotional system driving interpersonal communication between partners is a goal of neuroscience-based couple psychotherapy.

## 4. Cognitive psychopathology and psychotherapy

Cognitive defect varies to some extent in psychiatric disorders (Millan et al., 2012). However, it is still unclear how cognitive deficits may limit the ability of patients to actually attend psychotherapy or how cognitive problems may preclude a positive response to treatment. Cognitive symptoms such as those related to frontal lobe damage provoke a series of cognitive (e.g., disturbances in attention, planning, rigidity, inertia, criticism, control, inhibition, and decision-making) and emotional deficits (e.g., apathy, abulia, anhedonia, impulsiveness, behavioral inadequacy, aggression, and sociopathy) can greatly impact psychological interventions in patients with neurological disorders (Robinson et al., 2019) or acquired brain injury (Thøgersen et al., 2022). Psychotherapists have to be aware of executive dysfunction hindering the therapeutic process as an impairment that can strongly restrain its effect (Diamond, 2013; Cozolino, 2017), specifically in the case of particular approaches designed for older adults because of diminished specific frontal skills reported in elderly (Goodkind et al., 2016).

In this context, cognitive psychopathology offers an original contribution to psychotherapy for an in-depth understanding of psychiatric disturbances by making available the broad patrimony inherited from modern clinical neuropsychology (Timpano Sportiello, 2008). In particular, it can contribute to improving psychotherapeutic practice by refining the diagnosis and the differential diagnosis, offering relevant information to be provided to psychiatrists in the case of patients following combined treatments (i.e., neuropsychopharmacology), and enhancing the reliability of the prognostic judgment, also in relation to psychosocial rehabilitation interventions (e.g., behavioral skills training in psychiatric disorders). Cognitive dysfunction predicts psychosocial impairment; thus, its assessment is fundamental for rehabilitation purposes, especially in the case of severe psychiatric diseases (Etkin et al., 2022).

Disturbances in autobiographical memory, reality testing, interpretation of others, and fragmentation of a person's thought as an individual apart from everyone else are the consequences of the disruption of the “Default Mode Network” (DMN) (Raichle and Snyder, 2007; Andrews-Hanna et al., 2014; Mak et al., 2017) and are present in anxiety, depression, PSTD, and schizophrenia (Broyd et al., 2009; Cozolino, 2017). Neural areas, including the medial prefrontal cortex, the midline regions of the posterior cingulate cortex, and the precuneus region of the parietal cortex that turned on/off only to self-related task engaging of an individual, have been recently shown to support the DMN (Raichle and Snyder, 2007; Mak et al., 2017; Buckner and DiNicola, 2019). These findings have sustained the hypothesis that it may be the neuronal basis of the *Self* (i.e., personal and social awareness and ability in differentiated cognition and perception) (Faustino, 2022). Moreover, the dorsolateral prefrontal cortex, which is considered the neural circuit of working memory, also enables us to pay attention to something in the *here-and-now* (Siegel, 2006). Results are fundamental in psychotherapy—especially in Gestalt therapy—because its normal functioning permits to place the basis for the correct understanding of present feelings, emotions, and interpersonal reactions as they occur in the ongoing treatment sessions with no or little emphasis on past experiences. In a few words, it allows patients to concentrate on a new way of behaving built together with the psychotherapist.

The cognitive psychopathology perspective proposes that cognitive dysfunctions, which are closely related to emotional and relational processes, may contribute to the development, maintenance, and recurrence of psychiatric symptoms (Seron and Van der Linden, 2000). Psychotherapists must be exercised in drawing specific conclusions from a deep investigation of patients, including their cognitive evaluation, with the aim of taking into account particular mechanisms associated with mental pathology during the treatment course. Cognitive impairment consisting of dysfunction in working memory, attention/executive functions, processing speed, and visual and verbal learning represents a core feature of schizophrenia that is present in 62%−98% of patients and has been described in the first psychiatric episode, in healthy close relatives and in persons at high risk of developing the disease, and both in ongoing and remission phases (Morozova et al., 2022). Anxious symptoms produce significant deficits in attention control efficiency, especially in inhibition and switching (Shi et al., 2019). Symptoms frequently complained by depressed patients include attentional lability, concentration difficulties, dysmnesia (e.g., forgetfulness and word-finding difficulty), problem-solving, decision-making, judgment, and mental slowness (Richardson and Adams, 2018), whereas cognitive impairment in bipolar disorder is not limited to the affective episodes but persist in euthymic phases and mainly pertain attention, executive functions, learning, memory, and psychomotor speed (Cipriani et al., 2017). Patients with obsessive–compulsive disorder are significantly impaired in visuospatial memory, executive functions, verbal memory, and verbal fluency (Shin et al., 2014). Poor executive or “top-down regulation” of appetitive (i.e., reward, incentive salience) or aversive (i.e., stress, negative affect) processes is recognized as a basic impairment in behavioral addiction and a potentially relevant target for intervention, too (Ramey and Regier, 2019).

Cognitive remediation (CR)—sometimes referred to as cognitive enhancement therapy—is an intervention targeting cognitive deficits using scientific principles of learning in order to improve functional outcomes and rehabilitation of life skills (Wykes, 2018). CR has been conducted in various psychiatric disorders. Empirical evidence supports its efficacy in schizophrenia (Vita et al., 2021). CR techniques have also been applied to eating and weight disorders, attention-deficit hyperactivity disorders, mood disorders, anxiety disorders, substance use disorders, and autism spectrum disorders (Kim et al., 2018). We encourage psychotherapists to practice cognitive remediation techniques before treatment, especially when patients are so ill, in order to train them in promoting reflection on their thinking styles and exploring new strategies for everyday life. Such an approach may decrement the high dropouts rate from treatment, increase engagement in the therapeutic alliance (Tchanturia and Hambrook, 2010), and provide an opportunity for extending cognitive improvements to everyday functioning (Wykes, 2018) before discussing about feelings and emotions representing the core elements of the psychotherapeutic process.

We also affirm that the relationship with the psychotherapist does not only work because of the emotional bond with the patient but also when the practitioner is able to assign him/her adequate cognitive tasks in real life by taking into account the cognitive biases specifically characterizing personal cognitive profile. Only a correct weighting of what the patient is able to transfer from the therapeutic session to practical life experiences provides a concrete possibility of change. This becomes particularly true for cognitive–behavior psychotherapy (CBT) using homework assignments in order to maximize its effect (Kazantzis et al., 2005). Quite recently, a neuroscience-informed cognitive–behavioral approach (i.e., “The Waves of the New ABCs”) was developed to describe a continuous processing of internal and external stimuli that results in emotions, behaviors, and thoughts using the ABCDE model (Field et al., 2015), too.

Finally, with specific regard to depression, cognitive psychopathology can offer useful indications for psychotherapeutic treatment. We stress that the medial and orbital prefrontal regions play a key role in mediating the interaction between affective states and cognition: Depressed patients show facilitation of performance when responding to stimuli with a negative emotional tone (Elliott et al., 2002). Remarkably, mood-congruent memory (MCM) represents a psychological phenomenon where emotional memory is biased toward contents affectively congruent with a past or current mood (Faul and LaBar, 2022). Especially in the case of the MCM phenomenon, we recommend psychotherapists to operate through normalizing a deranged cognitive control process, which determines that information with an adverse emotional tone is recorded and recalled more successfully, contributing, in turn, to mood deflection. In the opinion of the authors, since psychophysiology consists a part of clinical neuroscience (Rabavilas and Papageorgiou, 2003), it appears advantageous to monitor psychophysiological variations associated with the MCM phenomenon through the ambulatory technological system (Loeffler et al., 2013), in order to allow the patient a more comprehensive appraisal of his/her emotional involvement in remembering evocative stimuli. Biofeedback instrumentation is able to identify maladaptive physiological responding and recognizing of mind-body connections can further facilitate psychotherapeutic progress.

### Key points

- Cognitive deficits—especially executive ones—may preclude a successful psychotherapeutic treatment;
- The DMN may be recognized as the neuronal basis of the *Self* and its disruption is present in anxiety, depression, PSTD, and schizophrenia;
- Psychotherapists should assess their patients in terms of cognitive functioning according to the neuropsychological impaired profile typically reported in a specific psychiatric disease;
- CR therapy should be practiced by psychotherapists in the case of patients with serious illnesses prior to the treatment, with the final aim of a better therapy start;
- Homework assignments used in CBT should be formulated according to the potential cognitive deficits reported by patients in treatment; and
- Psychotherapists performing retrieval of memories during treatment should pay attention to the MCM phenomenon in the case of patients suffering from MDD.

## 5. The therapeutic process of human empathy

The mirror neuron system (MNS) represents a distributed network of brain cells that discharges when a primate performs an action or observes an action performed by a similar one (Jeon and Lee, 2018). The MSN plays an important role in imitative behavior, especially in deciphering others' actions. They were originally found in the macaque's brain on the ventral premotor cortex and inferior parietal lobule (IPL) (di Pellegrino et al., 1992; Gallese et al., 1996; Rizzolatti et al., 1996). Later neuroimaging studies revealed corresponding activations in some human brain districts, that is, the prefrontal motor cortex, the areas around the intraparietal sulcus, and the supratemporal sulcus (Jellema and Perrett, 2003; Puce and Perrett, 2003; Rizzolatti and Craighero, 2004; Fogassi et al., 2005). Remarkably, the IPL visuo-motor organization receiving visual information from the eyes and somatosensory information from the mouth, the hands, and the arms calls into question the evidence that it may represent the neural basis of the ability to understand the intentions of the actions performed by others (Fogassi et al., 2005).

Mirror neurons are not only involved in motor perception but also in interpersonal cognition (Baird et al., 2011), suggesting that people perceive emotions in others by activating the same emotional response in themselves (Gallese, 2003). Although no conclusive evidence for a “broken mirror theory” has been provided, it has been hypothesized that a neuropathological functioning of the brain structures associated with mentalization deficits may exist in some psychiatric disorders characterized by social-cognitive deficits such as schizophrenia (van der Weiden et al., 2015) and autism spectrum disorders (Cattaneo et al., 2007; Gallese et al., 2013). Deficits in social cognition would depend on the particular function investigated (e.g., social threat and facial recognition) and are associated with characteristic symptoms of specific personality disorders (Herpertz and Bertsch, 2014).

Beyond the MSN, the empathy-related processing largely used in the therapeutic relationship also involves the temporo-parietal areas, the prefrontal cortex, and the temporal poles as a neurobiological substrate of the ToM, referring to the metacognitive ability to infer another person's mental state from his/her experiences and behavior (Vogeley et al., 2001; Vogeley and Fink, 2003; Frith and Frith, 2007; Schulte-Rüther et al., 2007). Remarkably, the dorsolateral prefrontal cortex strongly participates in the empathic response through emotion regulation and perspective-taking, and such capacities are reflected by brain structural variations in psychotherapists. Domínguez-Arriola et al. (2022) have demonstrated that psychotherapists display a significantly thicker left dorsolateral prefrontal cortex on the A9/46d region than non-therapists and that it correlates with the Empathic Concern (EC) scale score but not with any of the other psychometric measures adopted in the study. The authors concluded that the greater thickness of this region could reflect a superior tendency to regulate one's affective state in a professional context that demands augmented empathic skills than other jobs.

Scientific evidence on human empathy research is consistent with Kohut's emphasis on the therapeutic understanding of the patient prior to the interpretation of his/her dynamics (Stone, 2005). The discovery of mirroring mechanisms generates interesting implications for psychotherapeutic practice. The patient has an innate and programmed capacity to internalize, embody, and imitate the state of another person, and through psychotherapy, he/she can discover himself/herself in the other's mind (Janiri et al., 2009). Accordingly, proximity to the patient (i.e., proxemics), bodily movements (i.e., kinesics), and paralanguage (i.e., a no-lexical component of speech) are fundamental dimensions in the therapeutic setting (Cappas et al., 2005) that have to be correctly managed by psychotherapists prior to discuss about feelings and emotions (Faustino, 2021).

The knowledge of the neurophysiology of human empathy is also fundamental in social behavior, and group psychotherapy can take advantage of it. Psychotherapy can use the power of relationships to help patients increase wellbeing and enhance interactive capacity in familiar and social environments, and group therapy offers a unique setting to this end. According to Badenoch and Cox (2010), the brain's capacity to change is sensitive to environments providing moderate emotional arousal, attuned interpersonal relationships, and experiences that disconfirm earlier implicit learnings. Furthermore, the practice carried out by a patient in a group setting allows them to observe from their own mind the minds of others and concurs in the ability to elaborate emotional states associated with past memories through increased integration between the middle prefrontal cortex and limbic regions, creating a broader sense of confidence and stability (Siegel, 2007) through discussion with the group. As the limbic region becomes more dominated by emotional resonance circuits than in the past, internal and behavioral reactivity to activating stimuli decreases, providing the possibility to experiment with control thanks to the group as a source of response regulation (Badenoch, 2008). According to Schermer (2010), beyond mirroring and identification among group members, therapist attunement and interpretation represent two relevant features of group psychotherapy. The therapist attends the non-verbal and paraverbal communication as an expression of bonding and mutuality that emerge in the group. Since mirror neurons can register shared intentions, goals, and emotions, they impact the ongoing cohesion, norms, affective tone, and objectives of each session. Moreover, when the group is a close-knit one, and the psychotherapist has been able to nourish it, members often feel as if they can anticipate what someone is going to say next in the session, and such an impression is therapeutic because it helps members to feel less alone with their troubles and tuned with each other.

Finally, we also stress that psychotherapy practice based on empathic resonance is highly relevant in re-experiencing distressing life episodes to significantly impact response prevention by restraining patients from the use of unhelpful coping mechanisms and by improving behavioral control. Furthermore, it can exert an emotional regulation upon anger, guilt, and anxiety intensity and provides reassuring experiences that can permanently modify the implicit coding of stressful events. This is particularly true in PTSD, which is characterized by trauma re-experience, emotional numbing, avoidance, and exaggerated arousal (Frans et al., 2005). According to Peri et al. (2015), *face-to-face* exposure to traumatic memories in a safe environment with the psychotherapist may improve the acquisition of emotion regulation leading the patient to create new associative brain networks. Through the use of such a technique, modulated emotional responses are mirrored back to the patient by the psychotherapist allowing him/her to re-experience painful emotions in a controlled manner.

### Key points

- The MNS and the ability of the ToM support therapeutic relationship, and psychotherapists should be aware of this;
- The patient can discover himself/herself in the psychotherapist's mind during treatment and better hold his/her negative emotions;
- Group psychotherapy supports empathic resonance among members able to strength attentional control over the limbic system reactivity; and
- The exposure technique to traumatic memories in PTSD should be done in a *face-to-face* manner between the patient and the psychotherapist in order to improve emotional regulation.

## 6. Neuroimaging and psychotherapy

Psychotherapy represents a well-established strategy for a large part of psychiatric disorders. Despite this, psychotherapeutic interventions do not equally work for all patients because mechanisms through which they may reduce symptomatology remain difficult to fully understand. However, with the advent of neuroimaging techniques, researchers have new tools to find biomarkers of brain functioning associated with psychotherapeutic treatment or recognized as outcome predictors. Neuroimaging of patients with psychiatric disorders has revealed variances from healthy individuals, such as differences in measures of regional cerebral blood flow (rCBF), changes in local blood oxygenation level dependent (BOLD), brain metabolites levels, and functional connectivity (Weingarten and Strauman, 2015). Functional imaging methods comprise several types of modalities, such as functional magnetic resonance imaging (fMRI), positron emission tomography (PET), and single-photon emission computed tomography (SPECT). With different advantages and limitations (Peres and Nasello, 2008), such techniques share two general approaches to the study of psychotherapy effectiveness, that is, the usage of neuroimaging in the pre/post-treatment and the identification at baseline of brain-based predictors of response to treatment (Weingarten and Strauman, 2015).

With regard to the first approach, neurobiological research has shown a shared neural circuitry of emotion dysregulation associated with anxiety and depression. Prefrontal cortical regions, including the anterior cingulate cortex, the dorsomedial and ventromedial prefrontal cortices, as well as dorsolateral and ventrolateral prefrontal cortices, provide a “top-down regulation” over limbic regions (i.e., the amygdala, the hippocampus, and the insula) reacting to emotional information (Fournier and Price, 2014). Psychotherapeutic techniques relying on cognitive mechanisms associated with frontal domains, such as logical reasoning (e.g., emotions labeling), problem-solving, cognitive reappraisal, cognitive restructuring, modification of patient's self-representations, and mindfulness (Frewen et al., 2008) can help in remediating the inefficiency of such a regulation, by modulating reaction to negative emotional stimuli (Fournier and Price, 2014). Specifically, research in mindfulness currently integrates theory and methods from eastern contemplative traditions, western psychology, and neuroscience and is based on neuroimaging techniques, physiological measures, and behavioral tests (Tang et al., 2015). In addition to this, complementary emotion-focused techniques, such as experiential focusing, systematic evocative unfolding, evocative experiential states, and relaxation techniques (e.g., diaphragmatic breathing and progressive muscle relaxation) can contribute to enhancing emotional soothing by a “bottom-up regulation” of the subcortical network, too (Faustino, 2022). In light of this, we highlight that a top-down and bottom-up integrated approach to the treatment of anxious or depressive symptoms is necessary to counteract the symptomatology reported by the patient. A very recent systematic review of fMRI studies examining the neural basis of CBT concluded that although anxiety and associated disorders are mediated by different neural circuitry, it can increase prefrontal control of subcortical structures (Brooks and Stein, 2022). In a pool of fMRI studies, it has also been reported that amygdala hyperactivation in PTSD is due to the ineffective inhibitory control by the medial prefrontal cortex (Stevens et al., 2013), while dysregulation in corticostriatal circuitry has been reported to describe the neuropathology of obsessive–compulsive and related disorders (Milad and Rauch, 2012).

With regard to the second approach, the use of neuroimaging to identify predictors of treatment outcomes in patients with psychiatric disorders has been rapidly increasing in the last few years (Weingarten and Strauman, 2015). Sectorial literature investigating predictive neuroimaging markers of psychotherapy response has specifically suggested that the anterior cingulate cortex, amygdala, and anterior insula emerged as potential markers in MDD and some anxiety disorders (Chakrabarty et al., 2016). Two resting-state PET studies found that CBT responders have lower pretreatment metabolic activity in the subgenual anterior cingulate cortex (Konarski et al., 2009; McGrath et al., 2013); a hypometabolism of the right anterior insula was also found as associated with the positive response to CBT (McGrath et al., 2013). Other resting-state functional studies showed an increased orbitofrontal cortex activity as associated with response to behavioral therapy in obsessive–compulsive disorder (Brody et al., 1998; Yamanishi et al., 2009). In a sample of patients with panic disorder, an improved response to exposure-based CBT was predicted by increased pretreatment activation in the bilateral insula and left dorsolateral prefrontal cortex during threat processing, as well as increased right hippocampal gray matter volume (Reineke, 2014). An fMRI study concluded that the excessive amygdala response to fear reflecting difficulties in managing anxiety reactions elicited during CBT might limit optimal response to therapy in PTSD (Bryant et al., 2008). Hyperactivity of higher-order visual areas as a reaction to emotional stimuli was associated with a response to CBT for social anxiety (Doehrmann et al., 2013; Klumpp et al., 2013). CBT response was also predicted by pretreatment activity in prefrontal regions and the amygdala in patients with a generalized social anxiety disorder (Klumpp et al., 2014). Recently, it has been reported that the resting-state pretreatment metabolic activity in the fronto-insular cortex may distinguish between patients likely to respond to psychotherapy, while high metabolic activity in the subgenual anterior cingulate cortex may be predictive of poor outcomes in MDD (Dunlop and Mayberg, 2014).

We conclude that research in brain imaging and psychotherapy may increase the availability of evidence-based standardized protocols for selected groups of patients affected by psychiatric disorders. Since psychotherapy requires a considerable amount of time and effort, having the means to foresee from the beginning whether a patient is likely to benefit from treatment could be of great clinical utility, and neuroimaging represents a promising method to this end. However, research in this area needs to acquire other evidence, and a certain amount of caution must be used with respect to the single patient application of brain imaging techniques which should be corroborated by comparison of altered neurophysiological patterns typically involved in the same clinical population.

### Key points

- Neuroimaging techniques can be used to find biomarkers of brain functioning associated with psychotherapeutic treatment or to recognize outcome predictors;
- A neural circuitry of emotion dysregulation is associated with anxiety and depression symptoms, and psychotherapists should perform bottom-up and top-down regulation techniques to better control them;
- Research in brain imaging and psychotherapy may increase evidence-based standardized protocols for selected groups of psychiatric patients in order to augment treatment response and cost-effectiveness of health outcomes; and
- Technical and statistical limitations of integrating single-case (functional) neuroimaging and psychotherapy should be considered by therapists.

## 7. Interpersonal neurobiology perspective for somatic symptoms and related disorders

Various definitions, including “psychosomatic symptoms,” “functional symptoms,” “subjective health complaints,” “somatization,” “somatic symptom distress,” and “bodily distress,” have been used to depict a person's suffering related to physical symptoms (Van den Bergh et al., 2017). Diagnostic categories of *Somatic Symptom Disorder* (SSD) in the *Diagnostic and Statistical Manual for Mental Disorders*, fifth edition (DSM-5) (APA, 2013), *Somatoform Disorders* in the 10th Revision of the *International Statistical Classification of Diseases and Related Health Problems* (ICD-10) (World Health Organization, 2004) or *Bodily Distress Disorder* in the ICD-11 (WHO, 2019) are currently used in psychiatry nosography to classify the suffering of a person with a significant focus on physical symptoms such as pain, weakness, and breathlessness to a level that results in significant distress and functional limitation, low quality of life, work participation, and social interaction. Physical symptoms may or may not be associated with a diagnosed medical condition; however, the emphasis is on excessive thoughts, feelings, or behaviors related to their monitoring. Bodily distress is higher associated with depression and anxiety than specific medical conditions with comparable symptoms and well-defined organic pathology (e.g., IBS vs. inflammatory bowel disease, FMS vs. rheumatoid arthritis) (Henningsen, 2022). Moreover, individuals with somatic symptoms and related disorders experience difficulty in accepting that their concerns are excessive and prefer consulting general medical services rather than mental health services, which result in increased healthcare costs. A meta-analysis found that approximately 30% of patients in primary care settings fulfill the criteria for somatic symptoms disorder, and up to 50% of them present with at least one physical complaint (Haller et al., 2015).

Somatic symptoms and related disorders are challenging to treat for psychotherapists (Weigel et al., 2020). Because patients with somatic symptoms and related disorders are notably heterogeneous with respect to the nature and origins of their problems, the ability to design tailored interventions represents a central feature (Luyten and Fonagy, 2020). In the opinion of the authors, a neuroscience-based psychotherapy approach to such disorders should be performed within the framework of interpersonal neurobiology (IPNB), a scientifically grounded theory developed by Siegel (1999) as a field combining a wide array of science branches. The IPNB addresses three fundamental aspects of life, that is relationships (i.e., the sharing of energy and information flow), the brain (i.e., the embodied mechanisms of energy and information flow), and the mind (i.e., an emergent self-organizing activity of the brain regulating the flow of energy and information). Siegel stated that *A healthy mind is a mind that creates integration within the body and its brain* (…) (Siegel, 2019, p. 229). “Integration” represents the basis of harmony in human beings and is essential for their health. The IPNB perspective supports the hypothesis that mental disorders are both an outcome of blocked integration and result in further impairments to integration (Siegel, 2012). The integration or linking of differentiated parts of a system can be seen as the fundamental process of wellbeing and appears to be at the core of emotional regulation. Neural integration, enabling differentiated areas to communicate effectively to be part of a functional whole, can promote emotional regulation (Siegel, 2019). To this end, the “vertical integration” invoked by the author (Siegel, 2006) includes body-proper sensations, brainstem, limbic circuits, and middle prefrontal cortex structures. In the opinion of the author, this can be particularly relevant for somatic symptoms and related disorders treatment in order to transform a disconnected way of living into a more integrated personal identity. We think that mindful awareness training as a form of internal attunement (Siegel, 2012) can constitute an example of an intervention from which such a kind of mental disorder may benefit. Reducing physiological arousal and interoceptive hypervigilance through relaxation and exploring emotional control, experiences, expectations, beliefs, and illness behavior, as well as correcting catastrophic misinterpretations of somatic sensations (Van den Bergh et al., 2017; Henningsen, 2022) during psychotherapeutic treatment can further enhance the vertical integration.

### Key points

- The IPNB developed by Siegel (1999) may explain in more detail the need for integration between the body and the mind for subjective wellbeing;
- A “vertical integration” of body-proper sensations, brainstem, limbic circuits, and middle prefrontal cortex structures supported by specific techniques may aid the treatment of somatic symptoms and related disorders.

## 8. Discussion

According to previous attempts to point out neuroscience-based psychotherapy principles (Cappas et al., 2005; Faustino, 2021), the traditional dualism between brain and mind is no longer tenable. In the near future, we wish that neuroscience-based psychotherapy can contribute to the unification of its fragmented field, given that each specific school approach explains only 8% of the variance of the results and the most important common factor called into question to explain the patient's change after treatment is the therapeutic alliance (Wampold and Imel, 2015). Nearly 75% of patients commonly receiving psychotherapy improve after treatment compared to those who do not receive any treatment or ameliorate on their own (Jiménez et al., 2018). Moreover, psychotherapy—without difference in schools approach—is comparable in effectiveness to medication and has no relevant side effects (Leichsenring et al., 2022).

Memory is a fundamental trait of adaptive behavior, and it is never the same as itself. Starting from studies exploring molecular and cellular mechanisms underlying long-term memory formation and reconsolidation, psychotherapists should be fully aware of the crucial role they have in redesigning the patients' narrative plot, with a significant impact on identity and psychopathological symptoms relief. The investigations in which biological markers have found their way into psychotherapy research are still rare to date. We think that this represents a promising area to be implemented in the near future, bypassing potential methodological limitations (Piotrkowicz et al., 2021) as in the case of BDNF detection that should be added as a supplement to symptom scales commonly used to analyze psychotherapeutic effects. Since their potential to provide information about a therapeutic alliance that cannot be only derived from self-report questionnaires, salivary oxytocin/cortisol should be collected repeatedly during the treatment course from both patient and therapist before the therapeutic session, in order to collect biological data able to better describe the special bond between patient and therapist.

As people use their cognitive abilities to engage in everyday life, cognitive psychopathology has to be considered a crucial aspect of individuals adhering to psychotherapy. Many psychiatric disorders include the disruption of some aspects of cognition, and these deficits may predispose individuals to psychopathology, constitute an early marker, sustain the disorder, and predict the likelihood of functional recovery and successful rehabilitation. As a result, cognition and associated neural circuitry should be recognized as a key target of treatment by psychotherapists.

According to Bonini et al. (2022), studies of mirror mechanisms will lead to new research avenues in neuropsychiatric conditions in the near future. We are also confident in the increasing use of neuroimaging techniques to refine our understanding of both the outcome and process of psychotherapy, inform practitioners about evidence-based methods for specific psychiatric disorders, and help researchers to better define treatment protocols. In the opinion of the authors, an in-depth study of the implications of the dopaminergic mesocorticolimbic system of patients is really promising, with the aim of depicting the process of motivation to change as an intrinsic lever of the psychotherapeutic process. To this end, we think that brain imaging can play a relevant role, too.

Professionals must have timely access to information for optimal care implementation and a promising area of research based on advancements in MRI techniques (e.g., diffusion tensor imaging, DTI; BOLD fMRI signals from different brain regions) referred to the “connectome,” will permit researchers to shift attention from discrete brain areas to networks of brain regions supporting psychological dysfunction (Weingarten and Strauman, 2015). Systematic validation of biomarkers for independent clinical populations and integration with clinical data can augment their value for predicting psychotherapy outcomes. However, initial neuroimaging findings should be replicated in larger clinical populations and across a range of psychiatric disorders avoiding clinical decision-making on single cases due to the person's complexity (e.g., the potential presence of comorbidity) and limited generalizability of results.

Finally, we want to point out that by a neuroscience-based view of psychotherapy, the brain should be properly recognized as an interpersonal and a historical organ along the drawn lines of social neuroscience or neurophenomenology (cf., Varela, 1996; Cacioppo et al., 2002; Fuchs, 2003). This can stem a potential drift toward a more accurate knowledge of somatization and mind–body connection.

## 9. Conclusion

We have explored several paths regarding the neurobiological mechanisms through which psychological changes occur to sustain the future development of psychotherapy based on brain functioning and modeling. The latest discoveries in the neuroscientific field have shed light on the means by which psychotherapy proves to be a successful practice. For its part, psychotherapy can contribute to neuroscience by making available data from an accurate clinical activity that links the semeiotic investigation to the uniqueness of the patient and that bases its workout on the relational dimension of change, i.e., the therapeutic relationship. We also speculate that a neuroscience-based approach can really change and ameliorate our current psychotherapeutic interventions starting immediately by integrating in clinical practice patients' evaluation of cognitive domains mainly involved in the manifested mental disorder, top-down regulation techniques over the limbic system (i.e., emotions labeling, problem-solving, cognitive reappraisal, cognitive restructuring, mindfulness, and modification of patients' self-representations) and bottom-up interventions addressing somatosensory features of unresolved trauma (cf., Odgen et al., 2006) as well as “memory therapeutics” (i.e., accelerated resolution therapy, rewind techniques, cognitive restructuring, and imagery modification) able to redesign the narrative plot and the person's sense of the Self. Such improvements can be finalized after training of practitioners where necessary. Finally, according to a previous review suggesting that psychotherapy can influence the brain and behavior through the adaption of gene expression to the environment (Jiménez et al., 2018), we endorse the increasing consensus that it entails new learning in the context of an emotional relationship leading to epigenetic modifications after treatment.

## Author contributions

All authors listed have made a substantial, direct, and intellectual contribution to the work and approved it for publication.
